# Adolescent peer support for mental health problems: evaluation of the validity and reliability of the Mental Health Support Scale for Adolescents

**DOI:** 10.1186/s40359-023-01228-w

**Published:** 2023-06-30

**Authors:** Shurong Lu, Laura M. Hart, Anthony F. Jorm, Karen Gregg, Maxine Gross, Andrew J. Mackinnon, Amy J. Morgan

**Affiliations:** 1 Centre for Mental Health, Melbourne School of Population and Global Health, University of Melbourne, Parkville, VIC 3010 Australia; 2 School of Psychology and Public Health, La Trobe University, Melbourne, VIC 3086 Australia; 3 Black Dog Institute, University of New South Wales, Sydney, NSW 2031 Australia

**Keywords:** Mental health support scale for adolescents (MHSSA), Mental health first aid, Mental health literacy, Psychometrics, Adolescents

## Abstract

**Background:**

The *Mental Health Support Scale for Adolescents* (MHSSA) is a criterion-referenced measure of adolescents’ supportive intentions towards peers with mental health problems, which was developed for use in evaluations of adolescent mental health interventions, such as the teen Mental Health First Aid (tMHFA) program. The present study aimed to examine the validity and reliability of the MHSSA.

**Methods:**

A sample of 3092 school students (Mean ± SD: 15.9 ± 0.4 years old) and 65 tMHFA Instructors (the adult group with known expertise in tMHFA) completed the 12 items of the MHSSA. A sub-sample of 1201 students repeated the scale after a 3-4-week interval. Item concordance rates with the tMHFA Action Plan across helpful and harmful intentions scales were calculated. Scale reliabilities were assessed using agreement coefficients from a single test administration and test-retest reliability measured by intraclass correlation coefficients. The mean differences of MHSSA scores of students and Instructors were compared using independent samples t-tests, while convergent validity was tested via correlations of the scale with validated measures of confidence in providing help, social distance and personal stigma.

**Results:**

The average score of Instructors was significantly higher than that of students. The scale was positively associated with confidence in providing help, whilst negatively associated with social distance and dimensions of personal stigma. All scales of MHSSA had high agreement coefficients (all > 0.80) and fair to good test-retest reliability over 3–4 weeks.

**Conclusions:**

The MHSSA shows evidence of validity and reliability for use among adolescents for evaluating the quality of intentions to help peers with mental health problems.

**Supplementary Information:**

The online version contains supplementary material available at 10.1186/s40359-023-01228-w.

## Background

Poor mental health is a growing problem for adolescents. It impacts their life particularly in relation to education, employment, physical health and even life expectancy [1]. According to the recent World Mental Health Report, in 2019, around 14% of the world’s adolescents (aged 10–19 years) lived with a mental disorder [2], while about 13.9% of Australian children and adolescents aged 4–17 years experienced a mental disorder within 12 months [3]. Despite the first onset of mental disorders usually occurring in childhood or adolescence, treatment typically does not occur immediately [4], and delays in help seeking is a pervasive problem worldwide [5]. The most common barriers to accessing care for mental health problems include stigma, negative attitudes and perceptions, while mental health literacy acts as a facilitator [6].

### The teen Mental Health First Aid training program

Early interventions targeted at the whole population and groups of people such as adolescents have the potential to improve long-term mental health outcomes for individuals, families, and communities [7]. One such intervention is teen Mental Health First Aid (tMHFA), which is a novel, classroom-based training program for students aged 15–18 years. tMHFA is defined as the help an adolescent can give to a friend with a mental health problem, or a friend in a mental health crisis, until a reliable and trusted adult can take over [8, 9]. A three-session tMHFA training program has been disseminated through the global Mental Health First Aid (MHFA) program, which was developed in 2000 in Australia and aims to empower members of public to provide initial support to people experiencing mental health problems or crises until appropriate professional help is received or the crisis resolves [10].

The tMHFA program aims to help adolescents to better support their peers with a mental health problem [8, 9, 11], and its core course teaching is a five-point action plan based on the key messages for adolescents that were developed through a Delphi expert consensus method [12]. Specifically, the tMHFA Action Plan involves: (1) Look for warning signs; (2) Ask how they are; (3) Listen up; (4) Help them connect with an adult; and (5) Your friendship is important, which were shortened as “Look, Ask, Listen, Help, Your Friend” for an easy to remember format [9]. There is good evidence that the tMHFA intervention is an effective and feasible program for improving supportive behaviours towards peers, increasing mental health literacy and reducing stigma among adolescents [8, 11, 13].

### The development of the Mental Health Support Scale for Adolescents

While tMHFA focuses on providing support to others, many other mental health literacy programs in schools focus on personal help-seeking for mental health problems [14, 15]. Existing mental health literacy measures also often focus on personal help seeking rather than the provision of support, or help-giving, and are therefore not suitable for evaluating the impact of programs like tMHFA. Research on tMHFA aims to understand whether tMHFA training leads to better quality support towards adolescent peers and, ultimately, better outcomes for recipients of aid. However, to observe these effects, study designs need very large sample sizes, as well as a long follow-up period, allowing adequate time for support to occur and any impacts of that aid to be observed [16]. All of these requirements, however, can be challenging in terms of resources for research.

Measuring mental health first aid intentions may be an appropriate proxy for measuring actual help-giving behaviours. As per Ajzen’s Theory of Planned Behaviour, intentions to perform a given behaviour (for example, teenagers’ intentions to provide support to their peers experiencing a mental health problem) are hypothesised to predict performing the behaviour in the future [17]. As a general rule, the stronger the intention to engage in a behaviour, the more likely the behaviour is to occur [18]. Consistent with this, Yap et al. analysed two Australian national surveys of youth mental health literacy and found that the quality score of the mental health first aid intentions at baseline prospectively predicted the supportive behaviours offered to peers that were reported by participants at a 2-year follow-up [19]. Studies among adults similarly found that mental health first aid intentions can be used to predict their subsequent help-giving behaviours [20, 21].

Drawing on previous national mental health literacy surveys with youth [19] and preliminary evaluations of tMHFA [8], a criterion-referenced scale named the *Mental Health Support Scale for Adolescents* (MHSSA) was developed to measure the quality of mental health first aid intentions among adolescents, with the tMHFA Action Plan (as described previously) providing the criterion. This scale consists of items drawn from statements used in teen mental health first aid guidelines that were developed through a Delphi expert consensus method [12]. In contrast to commonly used norm-referenced measures, in which an individual’s performance is compared with that of others, criterion-referenced measures assess knowledge or skills against a set standard [22]. Such measures are used to determine whether an individual has reached a pre-defined level of competence or mastery of a skill, which would serve the purpose of the MHSSA measurement.

### Aims of the study

The present study aimed to examine the validity and reliability of the MHSSA in measuring adolescents’ intentions to support peers with mental health problems or crises. We hypothesised that the quality of mental health first aid intentions would discriminate between groups with and without expertise in tMHFA. To examine convergent validity, we assessed constructs that have been found to be related to mental health first aid intentions, including confidence in providing help, social distance and stigma [23–25]. Surveys of adolescents have found that confidence is associated with better quality mental health first aid intentions [23]. Similarly, higher levels of stigma and social distance are associated with inappropriate aiding intentions and actions [24, 25]. Therefore, we hypothesised that the quality of mental health first aid intentions would be positively correlated with confidence in providing help, but negatively with social distance and stigma towards people with mental health problems.

## Methods

### Participants and recruitment

This study used two sources of participants: senior students from 10 secondary schools (the targeted population that the MHSSA is designed for) who were naive to tMHFA and similar mental health interventions on help-giving or social support; and accredited tMHFA Instructors who are trained and qualified to deliver the tMHFA program in schools, as licensed by MHFA Australia (note: an Australian national not-for-profit organisation that develops, delivers and evaluates accredited mental health training programs). The instructors served as a referent expert group to confirm that each item was concordant (or not) with the tMHFA Action Plan and to test how well the scale discriminates between respondents with different levels of expertise in tMHFA.

Student participants were recruited from a randomised controlled trial (trial ID: ACTRN12617000633381) involving 10 government-funded secondary schools across the State of Victoria, Australia. For the current study, data was taken from students whose school was randomized to either the intervention group (where only the baseline measurement occasion was used), or to a control group (baseline and a second measurement occasions were used, and therefore allowing test re-test reliability analysis). All participants were given a Plain Language Statement and provided informed consent online. For student participants aged 14–18 years old, informed passive written consent was sought and obtained from their parents or guardians for study participation. Once a school had agreed to host the trial, all students at the Year 10 level were eligible to participate, unless a parent/guardian opted them out, or the student themselves didn’t provide their online assent for participation. Ethics approval for this trial was obtained from the Human Research Ethics Committee at the University of Melbourne (approval ID 1341238.4) and Victorian Department of Education (approval ID 2014_002268).

Accredited tMHFA Instructors were adults who have a high degree of competence in mental health first aid support to adolescents, because they undergo intensive training in the tMHFA course and are required to regularly deliver courses and undertake continuing professional development to maintain their accreditation. Accredited tMHFA Instructors based in Australia were recruited via direct emails or advertising posts to a Facebook group of Instructors administered by MHFA Australia. Surveys of tMHFA Instructors were approved by the Human Research Ethics Committee at the University of Melbourne (approval ID 2022-22917-26168-5).

## Measures

### The Mental Health Support Scale for Adolescents (MHSSA)

The MHSSA consists of 12 items measuring intentions to provide mental health first aid towards a hypothetical young person described in a vignette. Two vignettes were used in this study: one (John) depicting an adolescent with suicidal ideation and symptoms matching criteria for a depressive disorder according to the Diagnostic and Statistical Manual of Mental Disorders, 5th Edition (DSM-5) and International Statistical Classification of Diseases and Related Health Problems, 10th Revision (ICD-10) (American Psychiatric Association, 2013; World Health Organization, 1994); and the other (Jeanie) with symptoms matching criteria for social anxiety/phobia. These two vignettes were chosen because anxiety and depression represent the most common mental disorders occurring in adolescents of the target age range of MHSSA [3], and therefore present the scenarios (of developing mental health problems and crises such as suicidal distress) they are most likely to encounter in their peers. In addition, these vignettes had been used previously in national surveys of mental health literacy with adolescents [26, 27], and were well validated.

After the presentation of each vignette, the MHSSA asks *“If [John/Jeanie] were a friend I would…”* and presents a 5-point Likert response scale (1 = “*Never do this*”, 2 = “*Unlikely to do this*”, 3 = “*Not sure*”, 4 = “*Probably do this*”, and 5 = “*Definitely do this*”) for each of 12 items describing potential strategies for responding to the character in the vignette, for example, *“Suggest John tell a health professional about his problems (e.g. a counsellor, GP or psychologist)”.* The specific description of the two vignettes, as well as a copy of the MHSSA items, can be seen in Supplement 1.

Scale items were derived over the course of tMHFA evaluations [8, 13] and comprehensively revised during multiple workshops by a research group with expertise in tMHFA courses and/or general mental health first aid evaluations to ensure that they covered the spectrum of actions from the tMHFA Action Plan and reflected the key supportive strategies of tMHFA training. Given the importance of item selection and writing for the validity of a scale [22], a pilot survey of the MHSSA was conducted among its target population (i.e., adolescents aged 14–18 years, n = 23) to ensure that items were written at an age-appropriate reading level [8].

Scale items were designed to either be consistent with the tMHFA Action Plan (i.e., “helpful intentions”, n = 6, e.g., *Tell John/Jeanie I have noticed something seems wrong and I want to make sure s/he is okay*), or contrary to the plan (i.e., “harmful intentions”, n = 6, e.g., *Let John/Jeanie know I won’t want to be friends with him/her any more if s/he’s like this all the time)*. Helpful and Harmful items were counterbalanced to present an even mix of recommended actions and those that were not concordant with the action plan but appeared plausible. This was done to increase the scale’s potential to be sensitive to change as a result of training intervention, and to reduce problems with acquiescence and social desirability bias [28]. Additionally, this mix would make it possible to demonstrate improvements in recommended actions as well as reductions in unhelpful or harmful types of behaviours [9]. The label “harmful” was applied in this analysis due to the potential link of such items with harmful consequences or health impacts on recipients. However, it is important to point out that the endorsement of “harmful” items does not necessarily reflect any direct intent to harm, as these choices could reflect lack of relevant knowledge or failure to recall principles presented in the tMHFA training.

A full list of items in the original form as presented to participants are shown in Table 1. Items were given labels to aid analysis and reporting in the current study, and they were used to refer to items herein (see Table 1). It’s necessary to note that these labels were not presented to nor used by participants, who only saw the original items in full expressions.

**Table 1:** Scale items in original full expression, item labels, and the property they measured

Order	Original full expression of items	Item label for analysis^†^	Measured property
1	Invite John/Jeanie to hang out and do something fun with me.	Invite to hang out	Helpful intentions
2	Tell John/Jeanie I have noticed something seems wrong and I want to make sure s/he is okay.	Approach the person	
3	Suggest John/Jeanie tell a health professional about his/her problems (e.g. a counsellor, GP or psychologist).	Suggest telling a professional	
4	Suggest John/Jeanie tell an adult (other than a health professional) about his/her problems (e.g., parent or teacher).	Suggest telling an adult	
5	Ask John/Jeanie if s/he is thinking of suicide.	Ask about suicide	
6	Listen to John/Jeanie talk about his/her problems.	Listen to the person	
1	Tell John/Jeanie what s/he needs to do to fix his/her problems.	Tell the person how to fix	Harmful intentions
2	Ignore John/Jeanie because s/he is being attention-seeking.	Ignore the person	
3	Let John/Jeanie know I won’t want to be friends with him/her anymore if s/he’s like this all the time.	Unfriend the person	
4	Avoid talking about suicide because it might put the idea in John/Jeanie’s head.	Avoid talking about suicide	
5	Encourage John/Jeanie to take responsibility and deal with his/her problems on their own.	Encourage to deal alone	
6	Not do anything.	Do nothing	

^†^Items were given labels to aid analysis and reporting. These labels were not presented to nor used by participants, who only saw the original items in full.

### Other measures used for cross-validation of the scale

#### Confidence in providing help

Confidence in providing help to a peer with a mental health problem or crisis was assessed by the question “*If John/Jeanie was a friend, how confident would you feel helping him/her?*” with responses and scores of 1 = “*Not at all confident*”, 2 = “*A little bit confident*”, 3 = “*Moderately confident*”, 4 = “*Quite a bit confident*”, and 5 = “*Extremely confident*”. Higher scores indicate higher levels of confidence to help.

#### Social distance scale

The Social Distance Scale measures the desire to avoid contact with a person with a mental illness [27, 29]. Five items on desired social distance, the full-texts of which can be seen in Supplement 2, were measured on a 4-point Likert scale. Higher scores indicate greater social distance, and therefore stronger stigmatising attitudes. The measure has shown excellent reliability with α = 0.88 in community surveys of youth [27]. In the current study, McDonald’s omega (calculated based on the baseline data of students) for the scale was 0.95 for John and 0.96 for Jeanie. The validity of this scale is also supported by evidence that people with lower scores on social distance have more contact with people with mental disorders [30].

#### Personal stigma scale

The Depression Stigma Scale developed by Griffiths et al. [31] was modified by Hart et al. for use with the John/Jeanie vignettes in tMHFA [13]. Yap et al. [27] examined the properties of the scale in young people and reported that there were three distinct dimensions of “weak-not-sick” (i.e., viewing mental illness as a sign of personal weakness, rather than a medical illness), “dangerous/unpredictable” (i.e., seeing people with mental illness as dangerous or unpredictable), and “would not tell anyone” (i.e., would not tell anyone if they themselves had a mental health problem, reflecting personal stigma). The specific items of each dimension were presented in Supplement 2. In the current study, McDonald’s omega was 0.80 for John and 0.82 for Jeanie in the subscale “weak-not-sick”, and 0.71 for John and 0.84 for Jeanie in the subscale “dangerous/unpredictable” (note: the omega value for the subscale “would not tell anyone” was not calculated as it involves only one item).

### Data collection procedure

Eligible students (with passive parental consent) in Year 10 at secondary schools who had agreed to host the research were directed to an online questionnaire hosted by the SurveyMonkey platform (www.surveymonkey.com/mp/australia/), where they read a description of the research and provided electronic assent to indicate informed consent. All surveys were completed during regular class time and were supervised by teaching and research staff. Students completed the MHSSA (with both the vignettes of John and Jeanie being presented) and other measures described above. Basic demographic information (i.e., age, gender, language spoken at home) was also collected. Students took around 20–30 min to complete the baseline survey (which included additional measures not reported here) and were provided with an AUD 5 voucher as a gratuity for their attendance at a survey session, irrespective of their completeness the survey.

Eligible tMHFA Instructors were invited to take a separate online survey hosted by Qualtrics (https://www.qualtrics.com/au/), which included demographic questions (i.e., age, gender, the year they received their tMHFA Instructor accreditation, and the number of tMHFA courses they had delivered), as well as the MHSSA. Because mental health first aid as provided by adults [32] is different to the mental health first aid provided by adolescents [9], who are expected to take less responsibility for a peer’s wellbeing and to focus more strongly on getting an adult involved, Instructors were asked “*According to the tMHFA Action Plan, what do you think a teenager should do if they have a friend with a problem like John/Jeanie’s?*” when they were presented with the John and Jeanie vignettes. The Instructors were not asked to complete measures of convergent validity and as such their survey took approximately 10 min, and they received no compensation for participation.

### Statistical methods

#### Scale scoring and definition

As the MHSSA is a criterion-referenced measure, responses to each item were deemed either concordant or non-concordant with the tMHFA Action Plan (the referenced criterion, as described in the Methods). Based on having some level of intention to do it or avoid doing it among the adolescent sample (see Supplementary Table 1), concordant responses (scoring = 1) were defined as “*Probably do this*” or “*Definitely do this*” to a helpful item, OR, responding “*Never do this*” or “*Unlikely to do this*” to a harmful item (i.e., harmful items were reverse scored and higher scores on harmful intentions indicate better quality of intentions), as marked in Table 1. Otherwise, a response was defined as “non-concordant” (scoring = 0). Concordant rates for each scale item were calculated as the percent of cases giving responses that were concordant with the referenced criterion - tMHFA Action Plan.

Six scale scores were derived from the 12 MHSSA items. First, the sum scores for the two intention scales (helpful vs. harmful) were calculated by summing scores across vignettes to give robust measures of intentions across presentations of anxiety or depression-suicidality in a peer. Next, scores for the separate helpful and harmful scales of each vignette were calculated, so as to allow exploration of any differences in supportive intentions according to the presentation of a peer’s mental health problem. Therefore, this analysis included 6 scale scores: Helpful intentions, Harmful intentions (averaging the sum scores across vignettes); and Helpful intentions (John vignette: depression-suicidality), Harmful intentions (John vignette: depression-suicidality), Helpful intentions (Jeanie vignette: social anxiety/phobia), Harmful intentions (Jeanie vignette: social anxiety/phobia), which used the sum score of the relevant 6 items in each vignette separately.

In cases where participants had 1–2 missing items on the MHSSA scale, the missing values were replaced with the average score of the available responses. This occurred in 6 cases for the John vignette and 23 cases for the Jeanie vignette. Participants who did not provide any response to the scale for a given vignette were excluded from the analysis of that particular vignette. This resulted in the exclusion of 755 cases for the John vignette and 807 cases for the Jeanie vignette, corresponding to exclusion rates of 19.6% and 21.0%, respectively.

For each of the six scale scores, potential scores ranged from 0 to 6, with higher scores indicating higher concordance with the tMHFA Action Plan, i.e., better quality mental health first aid intentions. The total score of the 12 items summing across harmful and helpful was not calculated, because items of helpful and harmful measure different constructs and a recent study among adults revealed that separate scales of recommended and not recommended actions (similar to the helpful and harmful intentions in this scale) are not highly correlated [33].

#### Scale reliability

Conventional internal consistency reliability indices such as Cronbach’s Alpha are not considered appropriate for criterion-referenced measures because they primarily reflect how a set of items are closely related as a group [34], rather than assessing the overall consistency of the measure in classifying items as concordant or non-concordant against a fixed criterion. Therefore, the agreement coefficient for ordinal ratings from a single test administration was used in this analysis to measure the internal consistency of the MHSSA. Agreement coefficient estimates were calculated using the tables provided by Subkoviak and agreement coefficients of 0.75 or above were considered acceptable [35]. Given that the MHSSA was designed to be used with adolescents, agreement coefficients were calculated using the student sample.

Test-retest reliability was evaluated by calculating intraclass correlation coefficients (ICCs) between baseline and post-training scores of students that were allocated to the control group and did not receive the tMHFA intervention. According to the guidelines of Cicchetti, reliability coefficients < 0.40 represent poor reliability, 0.40–0.59 fair, 0.60–0.74 good, and 0.75–1.00 excellent [36].

#### Construct validity

Convergent validity was assessed by calculating Pearson correlation coefficients and their 95% confidence intervals (CIs) between scores averaged across vignettes of the scale and confidence in providing help, social distance and stigma dimensions.

The concordant rates of individual scale items and the mean and standard deviation (SD) of scale scores were calculated for both the samples of students and Instructors. The difference in concordance rates of the two groups were tested using chi-square tests, with *p*-values reported. For scale scores, Cohen’s d (note: a standardized effect size for measuring the difference between two group means, with values > 0.8 meaning a large effect size) and their 95% CIs were also reported. Two groups of participants with the lowest 10%, or the highest 10%, average scores across vignettes on the helpful/harmful scale of the MHSSA were extracted and their average scores across vignettes on validated measures (i.e., confidence in providing help, social distance and personal stigma) were compared using independent samples t-tests.

All analyses were conducted using Stata (version 17.0, College Station, Texas: StataCorp LLC), with a two-sided *P* value < 0.05 considered as statistically significant.

## Results

### Participant characteristics

There were 3094 students included in the analysis, with an average age of 15.9 years (SD = 0.4, range 14.5–18.4). About half of the students (49.0%) self-reported as female, two-thirds (68.4%) came from schools in metropolitan areas, and 86% of students spoke English as their first language. By vignette, 3092 students were included in the analysis for the John vignette (depression-suicidality) and 3040 for the Jeanie vignette (social anxiety/phobia), due to missing in responses. For the test-retest reliability analysis, the numbers were 1201 for John and 1149 for Jeanie, respectively.

There were 82 accredited tMHFA Instructors who gave their consent for participation. One was excluded from analysis due to not being Australia-based, 16 for missing more than 2 scale items on both vignettes and an additional 7 for failing to complete the Jeanie vignette. Therefore, the sample size for the Instructor analysis was 65 for the John vignette (female 48%, aged 49.0 ± 9.7 years) and 58 for the Jeanie vignette (female 44%, aged 49.1 ± 9.6 years). Instructors reported receiving their accreditation between 2010 and 2021 and 76.4% of them had 2 or more years of experience in tMHFA; on average, these Instructors had delivered 19 tMHFA courses (SD = 15, interquartile range 2–34).

### Concordance with the tMHFA Action Plan

The concordance rate of individual items for samples of students and Instructors is presented in Table 2 by scale. For the student sample, mean scores were on average lower for the harmful intentions scale (suggesting lower concordance with the tMHFA Action Plan) than for helpful intentions. Among the students, the item concordance rate was over 80% for 8 items in the John vignette and 6 in the Jeanie vignette, and several of these high concordance items (e.g., “*Listen to the person*”, “*Invite to hang out*” and “*Ignore the person*”) had a percentage that was close to or even higher than 90%. In comparison, the concordance rates of some items were particularly low, such as the item “*Tell the person how to fix*” (13.7% for John and 23.5% for Jeanie, respectively). The concordance rates for the two items related to suicide – “*Ask about suicide*” and “*Avoid talking about suicide*” – were lower than 30% for both vignettes, indicating poor suicide literacy among the students. Analysed by vignette, concordance rates of items were overall higher for John than for Jeanie. The difference between the two vignettes in the rate of concordance was over ± 9% in some items. For example, a much higher percentage of students reported concordance with the items “*Suggest telling a professional”*, and “*Suggest telling an adult”* in response to the John but not the Jeanie vignette. 15.8% versus 28.3% of students reported the concordant response to the item “*Avoid talking about suicide*” when presented with the John versus Jeanie vignettes respectively. That concordance was higher for Jeanie suggests that students were more likely to (endorse) avoid talking about suicide with John, despite (or perhaps because) John presented with overt suicide risk.

**Table 2:** Concordance rate of items and agreement coefficient by helpful/harmful scale

Scale	Item (by label)	Concordance rate (%)		Agreement coefficient^‡^
		Students	Instructors	
Helpful intentions^†^				0.87
Harmful intentions^†^				0.93
Helpful intentions (John vignette: depression-suicidality)	Invite to hang out	89.8	87.7	0.80
	Approach the person	91.3	98.5	
	Suggest telling a professional	81.3	98.5	
	Suggest telling an adult	84.0	100.0	
	Ask about suicide	29.6	95.4	
	Listen to the person	90.8	95.4	
Harmful intentions (John vignette: depression-suicidality)	Tell the person how to fix	13.7	86.2	0.92
	Ignore the person	89.5	98.5	
	Unfriend the person	88.5	98.5	
	Avoid talking about suicide	15.8	96.9	
	Encourage to deal alone	69.1	98.5	
	Do nothing	91.0	98.5	
Helpful intentions (Jeanie vignette: social anxiety/phobia)	Invite to hang out	88.5	89.7	0.84
	Approach the person	84.8	100.0	
	Suggest telling a professional	65.1	98.3	
	Suggest telling an adult	74.6	100.0	
	Ask about suicide	19.9	69.0	
	Listen to the person	89.1	96.6	
Harmful intentions (Jeanie vignette: social anxiety/phobia)	Tell the person how to fix	23.5	94.8	0.87
	Ignore the person	88.9	100.0	
	Unfriend the person	86.9	100.0	
	Avoid talking about suicide	28.3	100.0	
	Encourage to deal alone	64.7	98.3	
	Do nothing	86.0	100.0	

^†^Scales of helpful/harmful intentions used pooled data across vignettes; Items of harmful intentions were reverse scored, so higher concordance rates indicate better quality of intentions.

^‡^Agreement coefficients were calculated based on the student sample.

For the Instructor sample, high concordance rates (ranging from 86.2 to 100.0%) were observed in all items, except for “*Ask about suicide”* to Jeanie (69.0%), which may be due to the absence of obvious suicidal signs reflected in this vignette. Compared to the student group, the group of Instructors exhibited significantly higher concordance rates on all individual items, except for “*Invite to hang out*” and “*Listen to the person*”, consistently across both vignettes. This result could be attributed to the fact that these two items already had high concordance rates among both groups (see Table 2).

Additionally, 95% of Instructors scored 5 or above on both the helpful and harmful intentions scales, while 50% scored 6 (the highest score) on helpful intentions and 75% on harmful intentions, respectively, suggesting that mastery would be indicated by a score of 5 or above.

### Reliability of the scale

Table 2 also presents the agreement coefficient of each scale, which ranged between 0.80 (for the helpful intentions depression-suicidality scale) and 0.93 (for the harmful intentions scale). Overall, the harmful intentions scale had larger agreement coefficients than the helpful intentions scale.

The test-retest reliability (measured in ICC) was similar for the helpful and harmful scales, which was 0.62 (95% CI 0.58–0.66) and 0.63 (95% CI 0.58–0.67), respectively. The test-retest reliability for the scale of individual vignettes was around 0.55 (ranged 0.54–0.56).

The correlation matrix of scale scores is shown in Table 3. There were large correlations between the John and Jeanie vignettes for both helpful intentions (*r* = 0.56) and harmful intentions (*r* = 0.58), supporting the utility of pooling across vignettes. There was also a moderate-to-large correlation between the scales of helpful and harmful intentions (*r* = 0.40, 95% CI 0.37–0.43).

**Table 3:** Pearson correlation coefficients and their 95% CIs between average scores across vignettes and the helpful/harmful scale score of either vignette*

	Helpful intentions	Harmful intentions	Helpful intentions (John vignette: depression-suicidality)	Harmful intentions (John vignette: depression-suicidality)	Helpful intentions (Jeanie vignette: social anxiety/phobia)	Harmful intentions (Jeanie vignette: social anxiety/phobia)
Helpful intentions^†^	1					
Harmful intentions^†^	0.40 (0.37–0.43)	1				
Helpful intentions (John vignette: depression-suicidality)	0.86 (0.86–0.87)	0.35 (0.32–0.38)	1			
Harmful intentions (John vignette: depression-suicidality)	0.35 (0.32–0.38)	0.87 (0.86–0.88)	0.34 (0.31–0.37)	1		
Helpful intentions (Jeanie vignette: social anxiety/phobia)	0.90 (0.90–0.91)	0.36 (0.33–0.40)	0.56 (0.53–0.58)	0.29 (0.25–0.32)	1	
Harmful intentions (Jeanie vignette: social anxiety/phobia)	0.36 (0.33–0.39)	0.91 (0.90–0.92)	0.28 (0.25–0.32)	0.58 (0.55–0.60)	0.35 (0.32-0.39)	1

^*^All *p* values for the correlation coefficients < 0.001.

^†^Scales of helpful/harmful intentions used the data pooled across vignettes.

### Construct validity

**Fig. 1:**
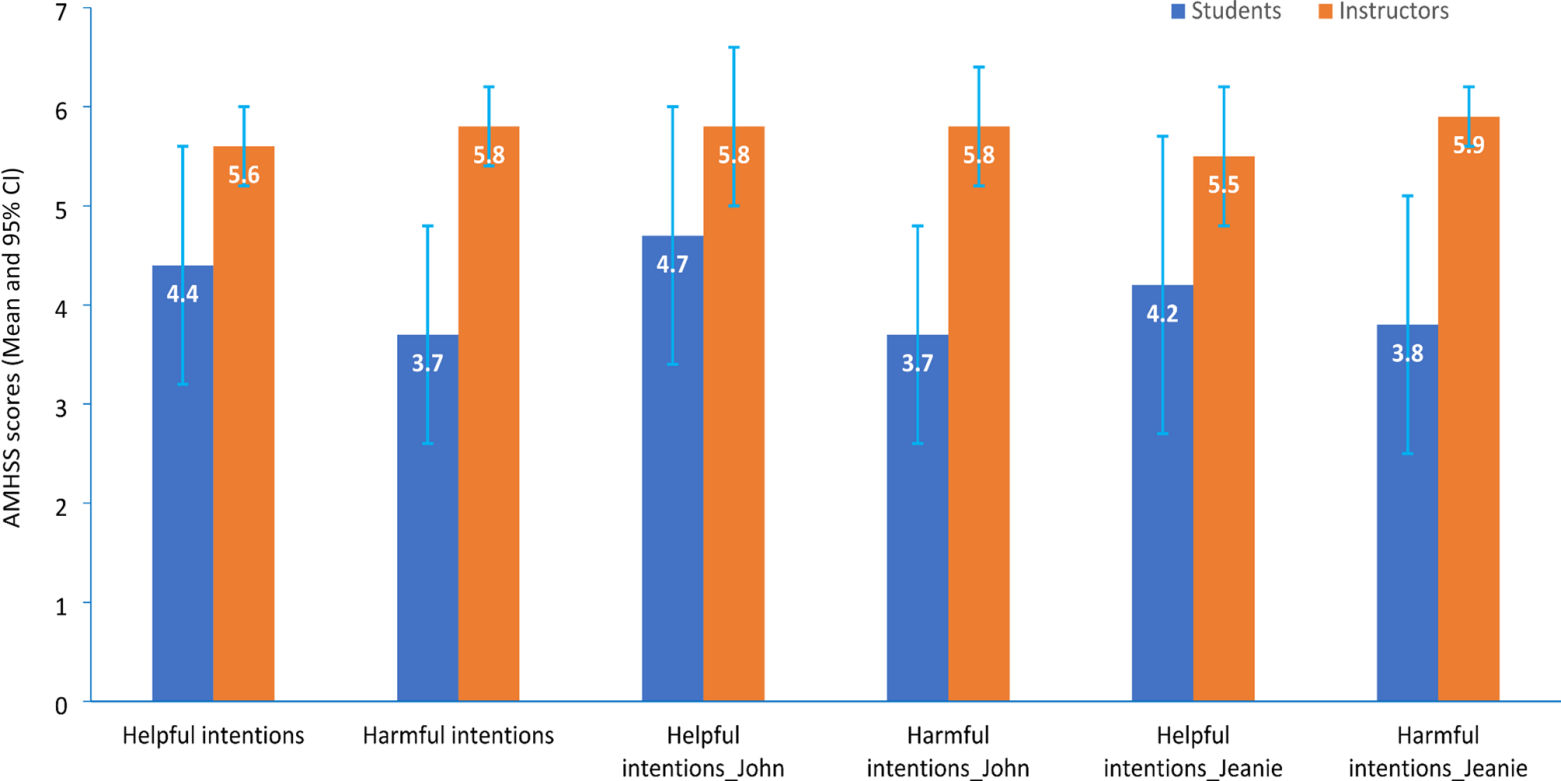
The scores of students and Instructors by helpful/harmful scale^†^ (Mean and 95% CI) ^†^The scale score of helpful/harmful intentions used data pooled across vignettes; Items of harmful intentions were reverse scored, so higher scores indicate better quality of intentions; Highest score for the helpful/harmful scale = 6 Bar for 95% CIs

Figure 1 illustrates the mean score of the helpful/harmful scale for samples of students and tMHFA Instructors, and Table 4 shows the difference of the two groups in mean scale scores and Cohen’s d values and 95% CIs. Consistent with our hypothesis, the mean scores in the Instructor sample were significantly higher than those in the student sample across scales. The differences in mean scores ranged from 1.1 to 2.2 (out of a total score of 6), with the largest differences observed for the harmful intentions scales (ranging from 2.1 to 2.2). All Cohen’s d values were larger than 0.8 (ranging 0.9–1.9), indicating large to very large effect sizes in the difference between students and Instructors.

**Table 4:** Differences of scale scores between students and Instructors

Scale	Difference^*^	Cohen’s d and 95% CI^‡^	
Helpful intentions^†^	1.2	1.0 (0.7–1.3)	
Harmful intentions^†^	2.1	1.9 (1.5–2.3)	
Helpful intentions (John vignette: depression-suicidality)	1.1	0.9 (0.6–1.2)	
Harmful intentions (John vignette: depression-suicidality)	2.1	1.9 (1.5–2.3)	
Helpful intentions (Jeanie vignette: social anxiety/phobia)	1.3	0.9 (0.6–1.2)	
Harmful intentions (Jeanie vignette: social anxiety/phobia)	2.2	1.9 (1.3-2.0)	

^†^Scales of helpful/harmful intentions used the data pooled across vignettes; Items of harmful intentions were reverse scored, so higher scores indicate better quality of intentions; Highest score for each scale = 6.

^‡^95% CI: 95% confidence interval.

^*^All *p* values for difference < 0.001.

**Fig. 2:**
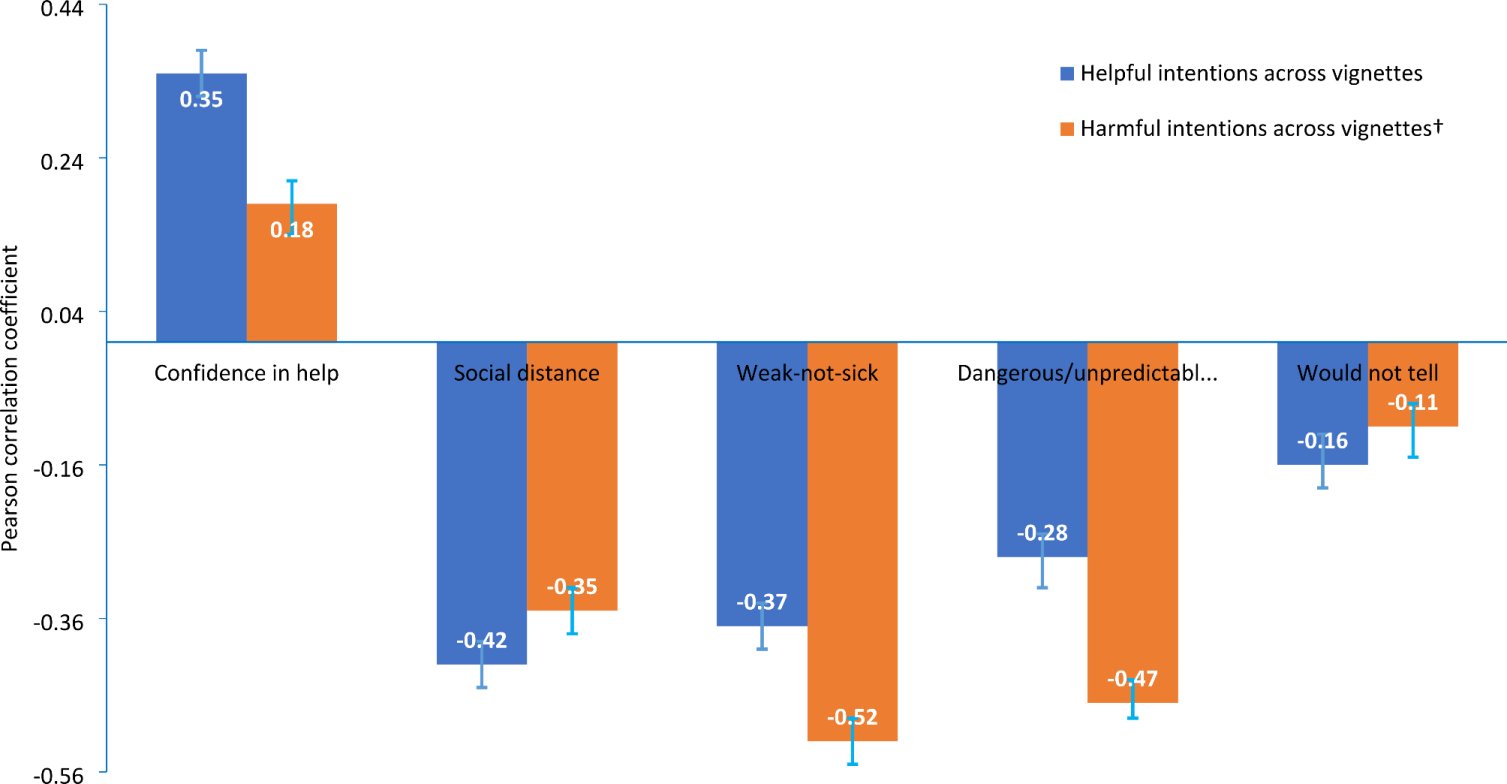
Pearson correlation coefficients and their 95% CIs between scores averaged across vignettes of the helpful/harmful scale and confidence in providing help, social distance and stigma dimensions ^†^To facilitate a comparison with the scores of helpful intentions, harmful intentions were reverse scored, with higher scores for better quality of intentions

As shown in Fig. 2, both the scores on the helpful and harmful intentions scales were positively correlated with confidence in providing help, and negatively correlated with social distance and all the three stigma dimensions (all *p* < 0.05), aligning with our hypotheses.

Compared to adolescents with the lowest 10% of MHSSA scores (either on the helpful or harmful scale), those with the top 10% of MHSSA scores had significantly higher confidence in providing help but significantly lower social distance and stigma, indicating good construct validity of the MHSSA (data not shown).

## Discussion

The MHSSA was developed as a criterion-referenced scale to measure the quality of mental health first aid intentions of adolescents towards their peers with mental health problems or crises. This study was the first to examine the psychometric properties of the scale in a large sample of adolescents, as well as a referent expert sample of tMHFA Instructors. Results show that the scale can discriminate the quality of mental health first aid intentions between respondents with and without expertise on tMHFA. The scale demonstrated high reliability when measured by agreement coefficients (above 0.80 for all scales) and fair to good test-retest reliability over 3–4 weeks (ICCs ranged 0.54–0.63). Evidence for convergent validity was provided by the correlations between intentions and confidence in providing help, social distance and stigma, which aligned with our hypotheses and were consistent with previous research [20, 26, 33]. These findings provide evidence for the validity and reliability of the MHSSA when used with adolescents in an Australian context.

The MHSSA measures helpful and harmful intentions in separate scales, aligning with previous evaluations of tMHFA [13]. These scales were moderately correlated but are important to measure separately, given that the tMHFA training could operate on each dimension in different ways. In the student sample, scores for harmful intentions were lower on average than those for helpful intentions, suggesting that tMHFA training may be especially important in shifting the intentions/behaviours that are not concordant with the criterion action plan. In addition, the extremely high concordance rate for two of the helpful intentions (“*Invite to hang out*” and “*Listen to the person*”), and the lack of significant difference in concordance rates between the groups of students and instructors, suggest that these items may be “too easy.“ It is possible that an empathetic or caring adolescent would be likely to respond correctly to these items, regardless of whether they had received tMHFA training or not. Therefore, these two items may be considered to be removed in future iterations of the scale.

In contrast, there were several items with very low concordance rates, particularly the two suicide-related items “*Ask about suicide*” and “*Avoid talking about suicide*”, which are likely due to the lack of relevant knowledge about suicide prevention among the student sample. Findings from surveys assessing suicide literacy of the adult public show that there are persistent misconceptions around asking about suicide (e.g., “*asking someone about suicide could make them start thinking about it*”) [37]. Thus, these intentions in particular are expected to be improved through the tMHFA training, given its focus on suicide prevention.

This study evaluated the scale against two hypothetical situations involving an adolescent with a mental health problem. There were some interesting differences in intentions towards the John vignette (depression-suicidality) compared with the Jeanie vignette (social anxiety/phobia). There was a much higher percentage of student participants suggesting John, rather than Jeanie, telling a professional (81.3% vs. 65.1%), or telling an adult (84.0% vs. 74.6%) about their problem. This indicates that social anxiety may be perceived as less deserving of adult intervention compared with depression-suicidality, despite anxiety-related disorders being the second most common mental health problems among Australian children and adolescents and being associated with high levels of burden and disability [3]. Our findings are similar to previous research, in which younger Australian adults (aged under 30) were found to provide less helpful responses to people with social anxiety as compared with other mental health problems [25]. Research in Australian adults has also shown drastically lower levels of recognition of social anxiety than depression (9% vs. 75%) [38]. Despite the differences across vignettes, our study showed that responses to the different vignettes were substantially correlated (*r* = 0.56 for helpful intentions and 0.58 for harmful intentions), and therefore, the scale is likely to be useful when applied to a broad range of common mental health problems and MHFA encounters among adolescents.

Although the scale was primarily designed for the evaluation of the tMHFA program, it has a much broader potential use in research settings to directly identify the general levels of supportive intentions towards adolescents confronting a mental health problem or crisis. The scales can also be used as a proxy for potential changes in helping behaviours, which are commonly challenging to assess in mental health intervention programs [16, 33], as well as an area of future research that is required of intervention programs for student mental health [39].

This study has several strengths. It is the first to comprehensively test the psychometric properties of a new criterion-referenced scale used for measuring the support that adolescents intend to provide to peers with mental health problems. The scale was evaluated with a large sample of adolescents and a sample of experts in tMHFA, which allowed for an evaluation on a range of indices of reliability and validity. Across a range of measures, the MHSSA was found to be sound and can be considered a useful tool for future evaluations of the quality of mental health first aid intentions among adolescents.

Nevertheless, the findings should be considered in light of study limitations. Firstly, this study examined the scale in only two of the most common mental health problems/crises among adolescents, so its validity for other mental health problems, or with different vignette genders (e.g., a female with depression-suicidality or a male with social anxiety/phobia), is unknown. Further evaluation is required to understand the validity of the scale in other mental health problems, such as attention deficit/hyperactivity disorders, substance use disorders and eating disorders, which are also common mental health problems among adolescents [3]. Secondly, test-retest reliability was measured based on the control group from the larger trial from which the sample for this study was drawn. The control group received an intervention in Physical First Aid training between the baseline and second measurement occasions. The similarity of tMHFA and Physical First Aid interventions (e.g., both recommend caring for others) may have led to lower reliability estimates. Future research may consider using a training-naive sample to examine re-test reliability to further understand how the measure operates over time. Finally, the study was conducted among adolescents residing in a single state within Australia, therefore its generalisability to adolescents in other regions, countries or cultures is unknown. The tMHFA program has been licensed and implemented in a range of nations, for example, United States, Wales, and the United Arab Emirates (https://mhfa.com.au/), so further use of the scale across a range of cultures and contexts is likely. Therefore, further culturally sensitive adaptation studies of the scale are warranted.

## Conclusions

Findings from this study provide evidence of the MHSSA as a reliable and valid measure of mental health first aid intentions for adolescents in an Australian context. The scale can be used to directly identify adolescents’ supportive intentions towards peers with a mental health problem or in a mental crisis. It can also be used as a proxy evaluative measure of adolescent mental health intervention programs targeting changes in providing supportive help to peers.

## Electronic supplementary material

Below is the link to the electronic supplementary material.

Supplementary Material 1: The Mental Health Support Scale for Adolescents.

## Data Availability

Raw data of this study cannot be shared due to ethical restrictions in accordance with the approved research protocol from the University of Melbourne and Victorian Department of Education under which the study was conducted, but de-identified data are available from the corresponding author on reasonable request.

## List of abbreviations

CI: Confidence Interval
DSM-5: The Diagnostic and Statistical Manual of Mental Disorders, 5th edition
ICC: Intraclass Correlation Coefficient
ICD-10: International Statistical Classification of Diseases and Related Health Problems, 10th revision
MHFA: Mental Health First Aid
MHSSA: The Mental Health Support Scale for Adolescents
SD: Standard Deviation
tMHFA: Teen Mental Health First Aid
