# Supplementary material for: Adolescent peer support for mental health problems: evaluation of the validity and reliability of the Mental Health Support Scale for Adolescents
Source: BMC Psychol. 2023 Jun 30;11:193. doi: 10.1186/s40359-023-01228-w (PMC10314445; doi:10.1186/s40359-023-01228-w)
Supplement: Supplementary file 1 — Supplementary Material 1: The Mental Health Support Scale for Adolescents. [file 40359_2023_1228_MOESM1_ESM.docx]

**Supplementary material**

# 1. The Adolescent Mental Health Support Scale

## John's Story (Depression-suicidality vignette)

John is a 16 year old who has been unusually sad and miserable for the last few weeks. He is tired all the time and has trouble sleeping at night. John doesn’t feel like eating and has lost weight. He can’t keep his mind on his studies and his marks have dropped. He puts off making any decisions and even day-to-day tasks seem too much for him. His parents and friends are very concerned about him. John says he will never be happy again and believes his family would be better off without him. John says he feels so desperate, he has been thinking of ways to end his life.

## Jeanie’s story (Social anxiety/phobia vignette)

Jeanie is a 16 year old living at home with her parents. Jeanie started at your school last year and you are the only friend she has made so far. She seems very shy and when you ask her why she doesn't make more of an effort, she says she would really like to make more friends but is scared that she’ll do or say something embarrassing when she’s around others. Although Jeanie’s schoolwork is OK she rarely says a word in class and becomes incredibly nervous, trembles, blushes and seems like she might vomit if she has to answer a question or speak in front of the class. At her house you have seen that Jeanie is quite talkative with her family, but becomes quiet if anyone she doesn’t know well comes over. She has stopped answering the phone and doesn't come to parties anymore. Jeanie says she knows her fears are unreasonable but she can’t seem to control them and this really upsets her.

## If John/Jeanie was a friend, I would…

|  | Never do this | Unlikely to do this | Not sure | Probably do this | Definitely do this |
| --- | --- | --- | --- | --- | --- |
| Tell John/Jeanie what s/he needs to do to fix his problems (harmful) |  |  |  |  |  |
| Invite John/Jeanie to hang out and do something fun with me (helpful) |  |  |  |  |  |
| Ignore John/Jeanie because s/he is being attention-seeking (harmful) |  |  |  |  |  |
| Let John/Jeanie know I won’t want to be friends with him any more if s/he’s like this all the time (harmful) |  |  |  |  |  |
| Avoid talking about suicide because it might put the idea in John/Jeanie’s head (harmful) |  |  |  |  |  |
| Tell John/Jeanie I have noticed something seems wrong and I want to make sure s/he is okay. (helpful) |  |  |  |  |  |
| Suggest John/Jeanie tell a health professional about his/her problems (e.g. a counsellor, GP or psychologist). (helpful) |  |  |  |  |  |
| Suggest John/Jeanie tell an adult (other than a health professional) about his/her problems (e.g. parent or teacher). (helpful) |  |  |  |  |  |
| Ask John/Jeanie if s/he is thinking of suicide. (helpful) |  |  |  |  |  |
| Encourage John/Jeanie to take responsibility and deal with his/her problems on his/her own. (harmful) |  |  |  |  |  |
| Listen to John/Jeanie talk about his/her problems. (helpful) |  |  |  |  |  |
| Not do anything. (harmful) |  |  |  |  |  |

^*^1. The notes of (harmful)/(helpful) were not part of the scale, but just used here to demonstrate the property of different items specifically for this study.

2. An extra item of “*Other (please specify)*” was used as 13^th^ item in the TeenAID trial, but was not included for analysis in this study, so it is not presented here.

# 2. Scale of Social Distance and Personal Stigma Scale

## Scale of Social Distance

The following questions ask how you would feel about spending time with John/Jeanie. *Would you be happy to:*

|  | Yes definitely | Yes probably | Probably not | Definitely not |
| --- | --- | --- | --- | --- |
| Develop a close friendship with John/Jeanie? |  |  |  |  |
| Go out with John/Jeanie on the weekend? |  |  |  |  |
| Go to John/Jeanie’s house? |  |  |  |  |
| Invite John/Jeanie around to your house? |  |  |  |  |
| Work on a project with John/Jeanie? |  |  |  |  |

## Personal Stigma Scale^*^

Please indicate how strongly you personally agree or disagree with each statement.

|  | Strongly disagree | Disagree | Neither agree nor disagree | Agree | Strongly agree |
| --- | --- | --- | --- | --- | --- |
| A problem like John/Jeanie’s is a sign of personal weakness (Weak-not-sick) |  |  |  |  |  |
| If I had a problem like John/Jeanie’s I would not tell anyone. (Would not tell anyone) |  |  |  |  |  |
| It is best to avoid people with a problem like John/Jeanie’s so that you don’t develop this problem.^#^ (Weak-not-sick) (Dangerous/Unpredictable) |  |  |  |  |  |
| John/Jeanie’s problem is not a real medical illness. (Weak-not-sick) |  |  |  |  |  |
| People with a problem like John/Jeanie’s are dangerous. (Dangerous/Unpredictable) |  |  |  |  |  |
| People with a problem like John/Jeanie’s are unpredictable. (Dangerous/Unpredictable) |  |  |  |  |  |
| People with a problem like John/Jeanie’s could snap out of it if they wanted. (Weak-not-sick) |  |  |  |  |  |

^*^(Weak-not-sick), (Dangerous/unpredictable) and (Would not tell anyone) were not part of the scale, but just used here to demonstrate the allocation of items to scale dimensions.

^#^ This item belongs to two dimensions for its cross loadings as observed in the property measurement study of the scale (*Yap MBH, Mackinnon A, Reavley N, Jorm AF: The measurement properties of stigmatizing attitudes towards mental disorders: results from two community surveys. Int J Meth Psych Res 2014, 23(1):49-61.*).
